# How Changes in Functional Disability Are Associated With Living Arrangements for Chinese Older Adults: Results From a Longitudinal Study (2008–2018)

**DOI:** 10.1111/ajag.70169

**Published:** 2026-04-21

**Authors:** Cai Xu, Yen‐Chang Chang, Yen‐Han Lee, Mack Shelley, Ke Li, Wenhan Guo

**Affiliations:** ^1^ Department of Social Medicine Heritage College of Osteopathic Medicine, Ohio University Athens Ohio USA; ^2^ Center for General Education, National Tsing Hua University Hsinchu Taiwan; ^3^ Department of Health Sciences College of Health Professions and Sciences, the University of Central Florida Orlando Florida USA; ^4^ Department of Political Science, Department of Statistics, and School of Education Iowa State University Ames Iowa USA; ^5^ Department of Social Work College of Health Sciences and Professions Athens Ohio USA; ^6^ Department of Social and Public Health College of Health Sciences and Professions Athens Ohio USA

**Keywords:** ADL, aged, China, living arrangements

## Abstract

**Objective:**

This study examined how changes in activities of daily living (ADL) and instrumental activities of daily living (IADL) relate to living arrangement transitions among older adults in China, and whether associations differ by urban and rural settings.

**Methods:**

Data came from the 2008–2018 Chinese Longitudinal Healthy Longevity Survey (CLHLS) of adults aged 65 years and older. Cox two‐state regression models assessed associations between functional transitions and living arrangement changes, stratified by community type.

**Results:**

Among 12,560 respondents, 81% lived with household members. Decline in ADL was associated with a higher hazard of transitioning to co‐residence (Hazard ratio (HR) = 1.16, 95% confidence interval (CI): 1.10–1.23) and a higher hazard of remaining in co‐residence (HR = 1.09, 95% CI: 1.01–1.17). The association with transitioning was observed in both urban (HR = 1.13) and rural (HR = 1.19) areas, while the association with remaining in co‐residence was significant only among rural older adults (HR = 1.23).

**Conclusions:**

Functional decline is longitudinally associated with a higher likelihood of transitioning to and remaining in co‐residence. This association is more pronounced in rural areas. Although ADL decline appears to be associated with subsequent changes in living arrangement, future research should explore its potential bidirectional nature to better inform community‐specific interventions.

## Impact Statements

**Practice Impact:**

Routine, community‐based monitoring of Activities of Daily Living can facilitate early intervention, support independent living and delay unnecessary institutionalisation. Strengthening caregiver support and programs that preserve functional capacity is essential to promote ageing in place and improve the quality of life for older adults.

**Policy Impact:**

Policies should expand home‐ and community‐based aged care, long‐term care insurance and caregiver support. Urban strategies should emphasise early detection and transitional care, whereas rural areas require sustained caregiver training, respite services and financial incentives, aligned with China's ‘home‐based, community‐supported, institution‐supplemented’ aged care model.

## 1 Introduction

Population ageing is a pressing global phenomenon that poses significant challenges to public health systems, social welfare programs and long‐term care services. China is experiencing rapid demographic shifts due to declining fertility rates and increased longevity. By 2025, nearly 323.38 million people in China were aged 60 years and above, representing 23% of the total population, with 223.65 million aged 65 years and above (16%) [1].

Functional health, often measured by difficulties in performing activities of daily living (ADL) and instrumental activities of daily living (IADL), is a key indicator of older adults' ability to live independently [2]. Activities of daily living include basic self‐care tasks (e.g., eating, dressing), while IADL covers more complex activities necessary for independent living (e.g., managing finances, transportation). Limitations in these domains reflect physical and cognitive decline and are associated with increased care needs and reduced quality of life. The prevalence of decline in ADL and IADL increases sharply with age. In China, 17% to 26% of community‐dwelling older adults aged 65 years and above experience ADL limitations, and 19% to 25% report IADL limitations [3, 4, 5, 6].

To meet growing aged care needs, China has adopted the ‘9073’ or ‘9064’ care models since 2009, whereby 90% of older adults prefer to age at home, 7%/6% receive community‐based care, and 3%/4% reside in institutions. This reflects a strong cultural preference for ageing in place [7], yet maintaining independent living is increasingly challenged by age‐related functional decline [8]. Declines in ADL and IADL may be linked to changes in living arrangements, reflecting response to emerging care needs [9].

Traditionally, aged care in China relies heavily on family members, especially married sons and their spouses [10], reflecting deep‐rooted cultural norms of filial piety and reinforced by legal obligations [11]. Formal long‐term care services, including nursing homes and community programs, remain underdeveloped and unevenly distributed, particularly in rural areas [12]. Although government initiatives such as the Star Light project aim to expand community‐based care, progress has been gradual and largely urban‐centred [13]. Public funding primarily supports highly vulnerable older adults without family and financial resources [14], while private paid care remains largely accessible only to wealthier families [15].

In contemporary China, older adults' living arrangements therefore remain predominantly family‐based. According to China's 2020 national census, 44% of older adults lived with a spouse only, 23% lived with both a spouse and children, and 17% lived with children; in contrast, approximately 12% lived alone, while institutional residence accounted for only 1%, far below the government's target of 3% [16]. In this context of limited institutional care and strong reliance on family co‐residence, most disabled older adults in community settings depend on informal care. Estimates from the *China Older Adult Health Report (2024)*, based on nationally representative data from the China Health and Retirement Longitudinal Study (CHARLS, 2021–2023), indicate that approximately 99% of disabled older adults receive informal care, primarily provided by family members such as spouses or adult children, whereas only about 1% receive formal care, defined as paid caregivers or professional care services [17]. This pattern aligns with global trends, as more than 80% of older adults worldwide, including those in China, prefer family‐based care for its familiarity and emotional support [18]. At the same time, a growing share of older adults are empty‐nest older adults, defined as those living alone or with a spouse only, without co‐residing adult children. In 2015, this group accounted for 52% of the older population (10% living alone and 42% living with a spouse) [19], and this proportion is projected to approach 90% by 2030 [20]. This demographic shift is accompanied by reduced availability of household‐based care and a higher prevalence of unmet care needs among older adults, particularly among those with disabilities or declining health. These trends highlight the importance of understanding how functional decline influences living arrangement decisions.

Previous studies have documented associations between living arrangements and functional status, including ADL and IADL disabilities among Chinese older adults. For example, living arrangements had been associated with slower ADL decline during the functionally independent stage, but showed less association once dependence begins [21]; living with or receiving care from relatives was associated with functional recovery and lower mortality risk [22]. Wang et al. [23] reported that living arrangements were significantly associated with both IADL and ADL disabilities among older Chinese adults. Wu et al. [24] found that among rural older adults in China, living alone or living with children without a spouse was linked to lower psychological well‐being, with ADL disability moderating the relationship by reducing the negative impact of living with children without a spouse.

However, most existing research focusses on static associations or single aspects of this relationship, without systematically examining how changes in functional status over time influence transitions in living arrangements. Moreover, longitudinal evidence stratified by urban and rural settings remains scarce. These gaps constrain understanding of the dynamic interplay between functional decline and living arrangement changes among Chinese older adults, particularly in the context of the rapidly increasing empty‐nest population.

To address these gaps, this study utilised longitudinal data from the CLHLS, a nationally representative dataset, spanning 2008–2018. Leveraging multi‐wave data allowed us to capture dynamic changes in functional disability and living arrangements over a decade. Importantly, we analysed urban and rural populations separately to identify disparities and heterogeneity in aged care contexts. Specifically, this study addressed two research questions: (1) How are changes in ADL and IADL associated with living arrangement transitions among Chinese older adults? (2) To what extent do these associations differ between urban and rural populations? The findings aim to deepen understanding of the interplay between functional decline and living arrangements, and help provide empirical evidence to inform targeted, context‐sensitive, and equitable aged care policies in China.

## 2 Methods

### 2.1 Data Source and Study Sample

We abstracted four waves of data, spanning a decade of study (2008–2009, 2011–2012, 2014 and 2017–2018) from the CLHLS, a publicly accessible and internationally recognised database established in 1998 through the collaborative efforts of the Duke Ageing Centre at Duke University, Durham, United States, and Peking University, Beijing, China. Since its inception, the CLHLS has conducted follow‐up surveys every 2–3 years. The CLHLS provides rich longitudinal data on health, functional status, sociodemographic characteristics and behavioural risk factors among older Chinese adults, including the oldest‐old, facilitating research on longevity and ageing trajectories in later life. During data collection, informed consent was obtained from all participants prior to the interviews. Subsequently, trained interviewers conducted face‐to‐face, home‐based interviews with randomly selected respondents across regions representing approximately 85% of the national population, completing a total of 113,000 interviews [25]. The CLHLS has maintained an overall response rate of over 90% across waves. The rigorous and systematic methodological framework employed by the CLHLS underpins the high quality of the dataset and ensures the robust reliability and validity of its measurement instruments. Detailed information of this dataset can be found in Zeng's comprehensive description [25].

To examine the association between changes in functional disability and living arrangements, we included adults aged 65 years or older who participated in at least two consecutive waves of the CLHLS between 2008 and 2018 and had no missing data for the variables of interest. The sample selection process was as follows: During the initial interview, respondents were required to provide complete responses to four core items: participant ID, interview year, ADL and IADL. Participants were retained only if they also provided complete responses to all relevant questions in at least one follow‐up interview, allowing us to assess within‐person changes in functional disability in terms of ADL and IADL over time.

**FIGURE A1:**
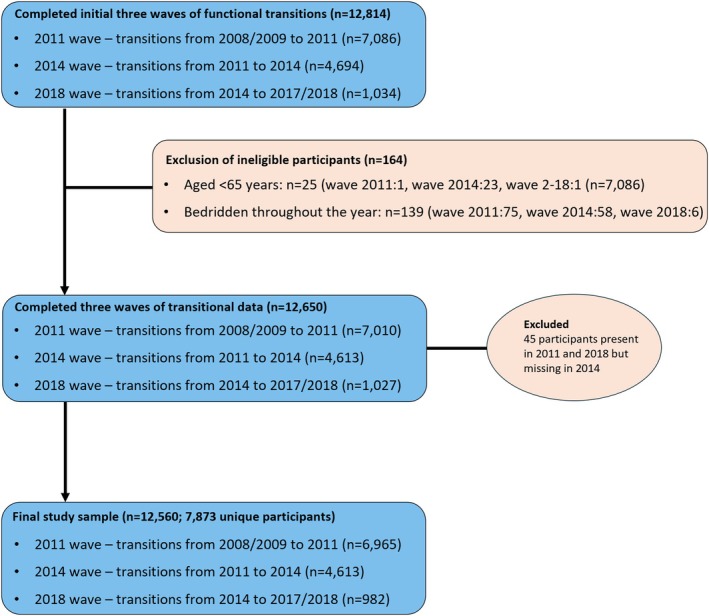
Flow diagram of study sample selection (*n* = 12,560).

Participants were excluded if they met any of the following criteria: (1) contributed data to both transition periods (i.e., 2008–2009 to 2011–2012 and 2014 to 2018) but were missing in the 2014 wave, as the extended interval may increase variability in living arrangements and functional status (ADL/IADL); (2) were confined to bed throughout the observation period, raising concerns about complete loss of ADL and IADL functions; or (3) were younger than 65 years. The final analytical sample comprised 12,560 person‐wave observations from 7873 unique individuals (see Appendix Figure A1).

The CLHLS had received prior ethics approval from the Institutional Review Boards (IRBs) of Duke University and Peking University (Approval No. IRB00001052‐13074) already. As this study involved secondary analysis of publicly available, de‐identified data with no personal information, it was not classified as human subjects' research and therefore did not require IRB approval [26, 27].

### 2.2 Outcome Variables

The living arrangement variable included two categories: (1) living with household member (s) and (2) not living with household member (s), which includes living alone or in an institution.

### 2.3 Primary Predictors

The two primary predictors were derived from participants' self‐reported disability status in the Chinese versions of the ADL and IADL scales. An ADL disability was defined as needing assistance with any of the following six basic activities: (1) bathing, (2) dressing, (3) eating, (4) transferring indoors, (5) toileting and (6) continence. Participants who reported being able to perform all six activities independently were categorised as ‘having no impairment’. Those requiring supervision, direction or personal assistance in one or more of these activities were classified as ‘*having impairment*’ [28]. An IADL disability was assessed based on participants' ability to carry out eight instrumental activities: (1) visiting neighbours, (2) shopping independently, (3) cooking, (4) washing clothes, (5) walking 1 km, (6) carrying 5 kg, (7) crouching and standing up three times and (8) taking public transportation. Participants who answered ‘yes’ to all eight items were considered as *‘*having no difficulty’, whereas those who reported ‘a little difficulty’ or being ‘unable to do’ any of these items were classified as ‘*having difficulty*’ [28].

To capture changes in physical functional disability over time, we created a three‐category transition variable: (1) from impairment/difficulty to no impairment/no difficulty, indicating improvement; (2) from no impairment/no difficulty to impairment/difficulty, indicating a decline; and (3) no change in ADL or IADL status across time points, indicating stability. This transition variable allowed us to track functional trajectories and assess their associations with subsequent living arrangement in older adults [29].

Specifically, changes in ADL and IADL were constructed between two consecutive survey waves (2008–2009 to 2011–2012, 2011–2012 to 2014 and 2014 to 2017–2018). Living arrangement status was then measured at the subsequent survey wave (2011–2012, 2014 and 2017–2018, respectively) and used as the outcome variable, ensuring that functional changes temporally preceded the observed living arrangement.

### 2.4 Covariates

We included several sociodemographic and health behaviour variables as covariates in the analysis. These comprised: age categorised as groups (65–80, 81–95, and older than 95 years), gender (female, male), educational attainment (no formal education vs. any formal education), community of residence (rural vs. urban) and marital status (married vs. others, including never married, divorced or widowed). Additionally, the number of chronic illnesses requiring treatment in the past 2 years was grouped as none, one to two and more than two. Life satisfaction was assessed using a self‐reported scale ranging from 1 (very poor) to 5 (excellent). Health behaviour variables included smoking, alcohol consumption and regular physical activity (all coded as yes/no). The model also adjusted for survey wave as a fixed effect, capturing data collection periods in 2011, 2014 and 2017–2018 (see Appendix Table A1 for full variable descriptions).

### 2.5 Statistical Analysis Strategies

We applied Cox regression models with two‐state transitions to examine how changes in physical disability relate to living arrangements. At each observation, living arrangements were categorised into two mutually exclusive states: co‐residing with household members and not co‐residing. In this study, we specifically examined two types of transitions: (1) from living separately to co‐residence, and (2) from co‐residing to continued co‐residence. Individuals contributed person‐time to a state‐specific risk set based on their current living arrangement, and transitions were treated as recurrent events, allowing multiple transitions across follow‐up periods. The probability of remaining in a given state was defined as the complement of the probability of transitioning to the alternative state at that time point. For example, the probability of remaining living separately was calculated as 1 minus the probability of transitioning to co‐residence, while the probability of transitioning from co‐residing to living separately was defined as 1 minus the probability of remaining co‐residing.

**FIGURE A2:**
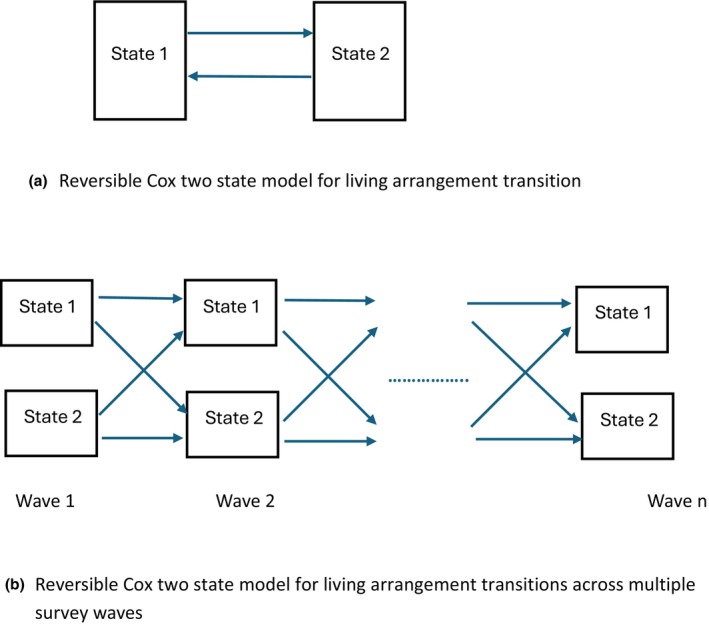
Two‐state reversible cox model for transitions in living arrangements State 1: Not with household member (s), State 2: With household member (s) (a) reversible cox two state model for living arrangement transition (b) reversible cox two state model for living arrangement transitions across multiple survey waves.

This modelling approach was selected because it accommodates the bidirectional and time‐varying nature of living arrangements, handles repeated measurements and accounts for the timing of multiple transitions. In contrast, conventional survival models assume unidirectional, irreversible events and cannot fully handle recurrent transitions. Compared with other longitudinal approaches, such as discrete‐time models or more complex multi‐state models, the Cox two‐state regression provides a flexible and interpretable framework well‐suited to our repeated observational data [29]. Detailed guidance on the implementation and interpretation of Cox multi‐state models can be found in Rademacher et al. (2022) [30], and the reversible states applied in this study are illustrated in Appendix Figure A2.

To explore potential differences across rural and urban settings, we incorporated an interaction term for residential context, given prior evidence of rural–urban disparities in physical function among older Chinese adults [31]. When the interaction between community of residence type and changes in physical disability reached statistical significance (*p* < 0.05), we conducted stratified analyses based on community type. To assess the robustness of this approach, we conducted sensitivity analyses using a more stringent threshold, defining impairment as reporting difficulties in two or more ADL/IADL items.

**FIGURE 1:**
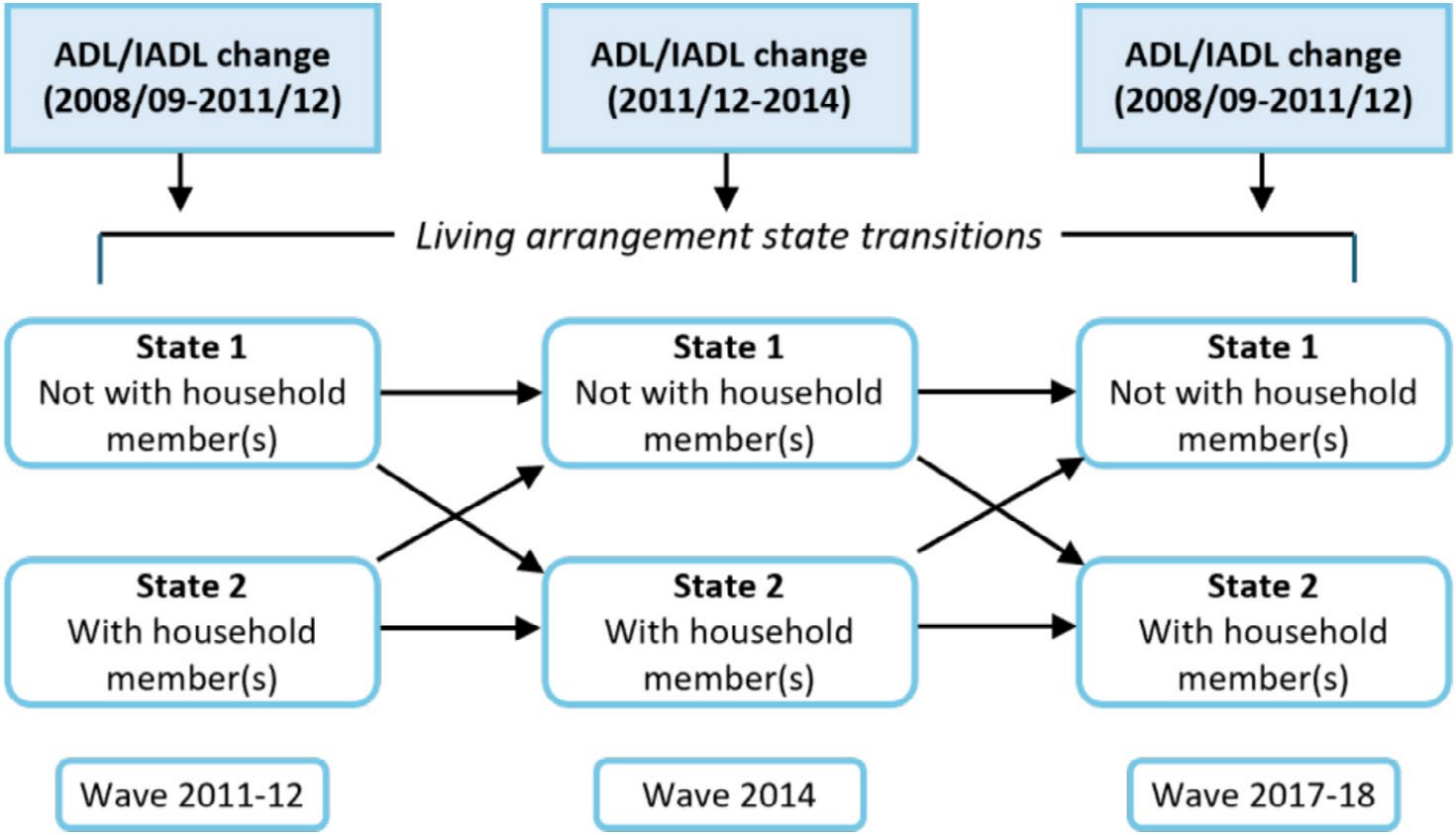
Two‐state living arrangement transitions by ADL/IADL changes.

Hazard ratios with 95% CIs were calculated. Statistical tests were two‐sided with significance threshold set at 0.05. All analyses were performed in R (version 4.2.1), utilising the ‘survival’ package for Cox modelling and the ‘psych’ package for descriptive statistics. Figure 1 illustrates the two‐state living arrangement transitions and functional disability changes, providing a visual summary of the analytic logic.

## 3 Results

### 3.1 Total Sample Characteristics

Table 1 summarises the characteristics of the analytic sample (*n* = 12,560 observations representing 7873 participants) across survey waves. Overall, 81% lived with household members. Participants had a mean age of 84.53 years (SD = 10.24); 52% were women and 54% had no formal education. The plurality was married (42%) and most lived in urban areas (53%). Regarding health behaviours, 81% were non‐smokers, 83% did not consume alcohol, and 65% did not engage in regular physical exercise. Additionally, 76% reported not having had a hospital‐treated chronic disease in the past 2 years. The average life satisfaction score was 3.79 (SD = 0.82).

**TABLE 1:** Sample characteristics by living arrangements among Chinese older adults: The CLHLS, 2008–2018 (*n* = 12,560).

		Living arrangement	
	Overall	With household member (s)	Not with household member (s)
	*n* (%)	*n* (%)	*n* (%)
Activities of daily living (ADL)transition			
No change	10,166 (81)	8115 (80)	2051 (84)
Impairment to no impairment (Improved)	443 (4)	336 (3)	107 (4)
No impairment to impairment (Declined)	1951 (16)	1665 (16)	286 (12)
Instrumental activities of daily living (IADL) transition			
No change	9046 (72)	7301 (72)	1745 (71)
Difficulty to no difficulty (Improved)	1042 (8)	822 (8)	220 (9)
No difficulty to difficulty (Declined)	2472 (20)	1993 (20)	479 (20)
Living arrangement			
With household member (s)	10,116 (81)		
Not with household member (s)	2444 (19)		
Age (mean, SD)	84.53 (10.24)	84.48 (10.46)	84.75 (9.29)
Age group			
65–80	4970 (40)	4105 (41)	865 (35)
81–95	5641 (45)	4371 (43)	1270 (52)
> 95	1949 (16)	1640 (16)	309 (13)
Gender			
Female	6584 (52)	5162 (51)	1422 (58)
Male	5976 (48)	4954 (49)	1022 (42)
Year of education (in years)			
No	6739 (54)	5235 (52)	1504 (62)
Yes	5,821 (46)	4881 (48)	940 (38)
Marital status			
Others	7304 (58)	5082 (50)	2222 (91)
Married	5256 (42)	5034 (50)	222 (9)
Community of residence			
Rural	5934 (47)	4709 (47)	1225 (50)
Urban	6626 (53)	5407 (53)	1219 (50)
Smoking status			
No	10,221 (81)	8187 (81)	2034 (83)
Yes	2339 (19)	1929 (19)	410 (17)
Alcohol consumption status			
No	10,411 (83)	8357 (83)	2054 (84)
Yes	2149 (17)	1759 (17)	390 (16)
Exercise status			
No	8111 (65)	6498 (64)	1613 (66)
Yes	4449 (35)	3618 (36)	831 (34)
Number of times suffering from chronic diseases that required inpatient treatment in the past 2 years			
None	9498 (76)	7567 (75)	1931 (79)
1–2	2618 (21)	2181 (22)	437 (18)
> 2	444 (4)	368 (4)	76 (3)
Life satisfaction (mean, SD)	3.79 (0.82)	3.82 (0.81)	3.65 (0.85)
Wave			
2011–2012	6965 (55)	5673 (56)	1292 (53)
2014	4613 (37)	3661 (36)	952 (39)
2017–2018	982 (8)	782 (8)	200 (8)

**FIGURE 2:**
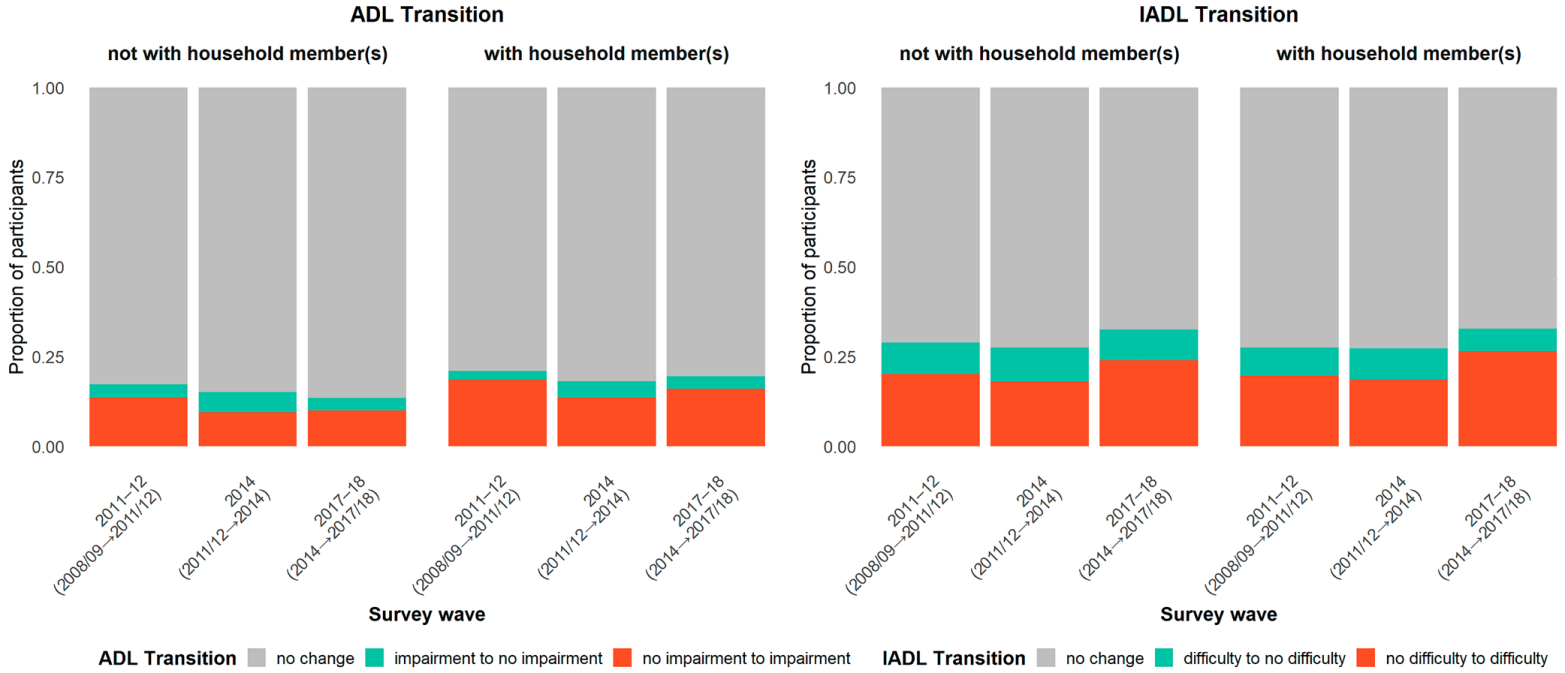
Transitions in ADL and IADL functioning by living arrangement across survey waves.

In terms of functional disability transitions, 16% of older adults experienced a decline in ADL, and 20% experienced a decline in IADL. However, the majority remained stable in both ADL (81%) and IADL (72%) status, as shown in Figure 2. Functional improvements were less common, reported by only 4% for ADL and 8% for IADL.

**FIGURE 3:**
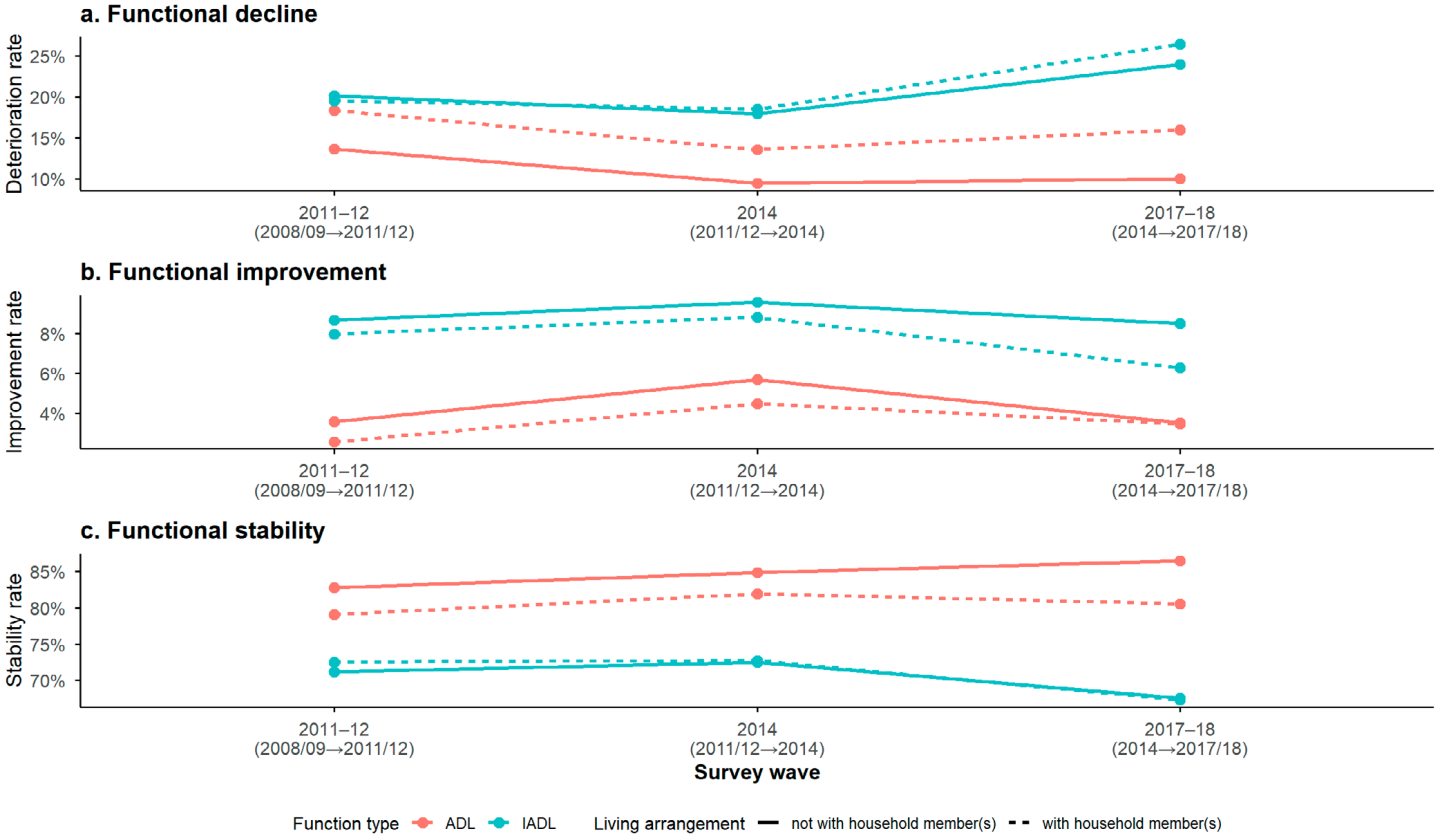
Functional transitions among older adults by living arrangement: Longitudinal trends in ADL and IADL from 2008 to 2018.

Figure 3 presents the distribution of living arrangement transitions across different types of functional status changes. Stability in both ADL and IADL was consistently associated with a higher likelihood of maintaining the same living arrangement. In contrast, those who experienced functional decline, especially in IADL, were more likely to live in co‐residence with household members. This trend was more evident in the 2017–2018 wave. Subsample characteristics stratified by living arrangement are also presented in Table 1. Comparative demographic characteristics between included and excluded participants are presented in Appendix Table A2.

### 3.2 Associations Between Changes in Physical Functions Status and Changes in Living Arrangement

As shown in Table 2, older adults who experienced a decline in ADL function (i.e., transitioned from no impairment to impairment) had significantly higher hazards of changing from not living with household member (s) to co‐residing (HR = 1.16, 95% CI: 1.10–1.23, *p* < 0.001) and of maintaining co‐residence with household member (s) (HR = 1.09, 95% CI: 1.01–1.17, *p* < 0.05).

**TABLE 2:** Associations of functional disability changes with living arrangements among Chinese older adults: Evidence from the CLHLS, 2008–2018.

	Change in living arrangement	
	From not with household member (s) to with household member (s)	From with household member (s) to with household member (s)
	HR (95% CI)	HR (95% CI)
ADL transition		
No change		
Impairment to no impairment (Improved)	0.91 (0.79, 1.04)	0.92 (0.80, 1.06)
No impairment to impairment (Declined)	1.16 (1.10, 1.23)***	1.09 (1.01, 1.17)*
IADL transition		
No change		
Difficulty to no difficulty (Improved)	1 (0.93, 1.07)	0.97 (0.88, 1.07)
No difficulty to difficulty (Declined)	1 (0.95, 1.05)	1.01 (0.95, 1.07)
Age (in years)		
65–80		
81–95	1.12 (1.07, 1.18)***	1.08 (1.02, 1.15)*
> 95	1.56 (1.45, 1.69)***	1.42 (1.30, 1.55)***
Gender		
Female		
Male	0.89 (0.84, 0.93)***	0.93 (0.87, 0.99)*
Formal education		
No		
Yes	1.05 (1.00, 1.10)	1.02 (0.96, 1.08)
Marital status		
Others		
Married	2.36 (2.24, 2.48)***	1.68 (1.58, 1.8)***
Community of residence		
Rural		
Urban	0.97 (0.93, 1.01)	1.04 (0.98, 1.09)
Smoking status		
No		
Yes	1.02 (0.97, 1.08)	0.99 (0.92, 1.06)
Alcohol consumption status		
No		
Yes	1.01 (0.95, 1.06)	0.95 (0.88, 1.02)
Exercise status		
No		
Yes	0.96 (0.92, 1.00)	1 (0.94, 1.05)
Number of times suffering from chronic diseases that required inpatient treatment in the past 2 years		
None		
1–2	1.02 (0.97, 1.07)	1.08 (1.02, 1.14)**
> 2	1.03 (0.93, 1.15)	1.03 (0.92, 1.16)
Life satisfaction	1.08 (1.06, 1.11)***	1.07 (1.04, 1.11)***

*Note:* *p < 0.05; **p < 0.01; ***p < 0.001.

Abbreviations: CI, confidence interval; HR, hazard ratio.

### 3.3 Associations Between Changes in Physical Function Status and Changes in Living Arrangements, Stratified by Community of Residence

Given the significant interaction between functional disability and community of residence (Appendix Table A3), we conducted stratified analyses, which revealed that the associations between changes in physical function and living arrangements differed by community, as shown in Table 3. Specifically, declines in ADL were significantly associated with an increased likelihood of transitioning from not living with household member (s) to co‐residing, both in urban (HR = 1.13, 95% CI: 1.05–1.22, *p* < 0.001) and rural (HR = 1.19, 95% CI: 1.10–1.28, *p* < 0.001) settings. Among rural older adults, ADL decline was also associated with higher hazards of remaining in co‐residence with household members (HR = 1.23, 95% CI: 1.10–1.39, *p* < 0.001).

**TABLE 3:** Associations of functional disability changes with living arrangements among Chinese older adults, stratified by community of residence: Evidence from the CLHLS, 2008–2018.

	Change in living arrangement among urban residents		Change in living arrangement among rural residents	
	From not with household member (s) to with household member (s)	From with household member (s) to with household member (s)	From not with household member (s) to with household member (s)	From with household member (s) to with household member (s)
	HR (95% CI)	HR (95% CI)	HR (95% CI)	HR (95% CI)
ADL transition				
No change				
Impairment to no impairment (Improved)	0.93 (0.79, 1.11)	0.84 (0.68, 1.03)	0.91 (0.76, 1.09)	1.04 (0.82, 1.31)
No impairment to impairment (Declined)	1.13 (1.05, 1.22)***	0.99 (0.9, 1.10)	1.19 (1.1, 1.28)***	1.23 (1.1, 1.39)***
IADL transition				
No change				
Difficulty to no difficulty (Improved)	1.01 (0.92, 1.12)	0.98 (0.85, 1.13)	0.95 (0.87, 1.05)	1.1 (0.94, 1.28)
No difficulty to difficulty (Declined)	0.99 (0.93, 1.05)	1.03 (0.95, 1.11)	0.99 (0.92, 1.06)	1.04 (0.93, 1.16)
Age (in years)				
65–80				
81–95	1.12 (1.05, 1.19)***	1.09 (1, 1.18)	1.12 (1.05, 1.20)***	1.08 (0.98, 1.20)
> 95	1.52 (1.38, 1.69)***	1.47 (1.3, 1.66)***	1.58 (1.43, 1.75)***	1.32 (1.12, 1.56)***
Gender				
Female				
Male	0.89 (0.83, 0.95)***	0.92 (0.83, 1.01)	0.89 (0.83, 0.95)***	0.93 (0.83, 1.04)
Formal education				
No				
Yes	1.05 (0.99, 1.12)	0.98 (0.9, 1.07)	1.05 (0.99, 1.12)	1.01 (0.91, 1.12)
Marital status				
Others				
Married	2.23 (2.08, 2.38)***	1.69 (1.54, 1.87)***	2.54 (2.37, 2.72)***	1.65 (1.47, 1.84)***
Smoking status				
No				
Yes	1.03 (0.96, 1.11)	0.99 (0.89, 1.11)	1.01 (0.95, 1.09)	1 (0.88, 1.13)
Alcohol consumption status				
No				
Yes	0.99 (0.92, 1.07)	0.96 (0.86, 1.07)	1.02 (0.94, 1.09)	0.96 (0.83, 1.10)
Exercise status				
No				
Yes	0.96 (0.9, 1.01)	1.03 (0.95, 1.11)	0.96 (0.9, 1.01)	0.98 (0.88, 1.09)
Number of times suffering from chronic diseases that required inpatient treatment in the past 2 years				
None				
1–2	1.05 (0.98, 1.12)	1.04 (0.97, 1.13)	1.04 (0.97, 1.11)	1.05 (0.94, 1.16)
> 2	1.12 (1, 1.26)	1.07 (0.92, 1.25)	0.98 (0.82, 1.16)	0.98 (0.76, 1.25)
Life satisfaction	1.07 (1.04, 1.11)***	1.06 (1.01, 1.11)*	1.11 (1.07, 1.15)***	1.08 (1.02, 1.14)**

*Note:* *p < 0.05; **p < 0.01; ***p < 0.001.

Abbreviations: CI, confidence interval; HR, hazard ratio.

Sensitivity analyses using a stricter cut‐off for functional disability (two or more ADL/IADL difficulties) yielded results generally consistent with the primary analyses, with only slight attenuation in effect sizes, supporting the robustness of our findings (Appendix Table A4, 1–3).

## 4 Discussion

Using nationally representative longitudinal data from the 2008–2018 waves of the CLHLS, this study examined how changes in physical function, measured by ADL and IADL transitions, affect living arrangement changes among older adults in China. By applying Cox two‐state regression models and stratifying by community of residence, we found that functional decline, particularly in ADL, was associated with a higher likelihood of transitioning to or maintaining co‐residence, with stronger associations observed in rural areas. This study is the first to explore urban–rural differences in the relationship between functional changes and living arrangements over time, highlighting the role of community context in shaping support needs for older adults.

In this study, we found that declines in ADL were significantly associated with older adults either transitioning to or maintaining co‐residence with household member (s), whereas changes in IADL were not significantly related to living arrangement transitions. This result may be because ADL, as a more severe form of function limitation [32], reflects fundamental self‐care abilities essential for basic daily functions such as eating, bathing and toileting. When these abilities decline, older adults are more likely to require substantial hands‐on support, making co‐residence with family a practical solution despite the high opportunity cost for caregivers [33], especially given the significant burden families face from the high prevalence and economic impact of late‐life disability.

In 2023, approximately 46.54 million older adults in China were living with disabilities, a number projected to rise to 97.5 million by 2050 [34]. According to the *China Elderly Health Report (2024)*, the annual average cost of formal care is 46,448 yuan (just under US$6800), rendering market‐based long‐term care unaffordable for many [34], As a result, the majority of disabled older adults in China rely on informal care rather than formal care [17]. While it is undeniable that cultural norms and filial piety influence the preference for co‐residence in aged care, this pattern is also driven by the lack of a systematic, sustainable and coordinated aged care system in China, characterised by limited capacity in community health centres, underdeveloped formal long‐term care service systems, insufficient services offered by social welfare institutions and inadequate support for family caregivers [35].

In contrast, IADL tasks, such as managing finances and transportation, are primarily cognitively and socially demanding but less physically intensive [36], and thus may not immediately necessitate changes in residential status or cohabitation patterns. Declines in IADL functioning can be manageable through external support, which may lessen the perceived need for residential transitions. Common support strategies include assistance from family or friends [37], home care services [38], assistive technologies [39] and home modifications [40]. These interventions are designed to preserve functional independence and enable older adults to remain safely in their preferred living environment. Timely support may mitigate the adverse effects of IADL decline and delay the need for more substantial changes in living arrangements, thereby facilitating ageing in place [41].

Of note, while changes in IADL were not significantly associated with living arrangement transitions in either urban or rural settings, declines in ADL showed distinct patterns by residential context. In urban areas, ADL deterioration was mainly linked to initiating co‐residence with family members, whereas in rural areas, it was related to both initiating and maintaining such arrangements. This divergence likely reflects enduring caregiving norms and limited availability of formal aged care services in rural communities [42]. Our findings were consistent with previous studies showing higher rates of co‐residence in rural areas and substantially lower access to formal care for disabled older adults in rural than in urban areas [33, 43].

Beyond the Chinese context, the patterns observed in this study contrast with those documented in many Western high‐income countries. In the United States, the onset of disability and rising care needs substantially increase the likelihood of admission to nursing homes or other long‐term care facilities, particularly when spouses or adult children are unavailable or unable to provide intensive care [44, 45]. In much of Europe, long‐term care reforms have instead prioritised ‘ageing in place’, expanding community‐based and home‐care services to help older adults remain in their own homes despite ADL/IADL limitations [46, 47]. As functional impairment progresses, support typically combines formal home‐care services with extensive assistance from family members and other informal caregivers; however, unmet care needs and support gaps remain widespread [48]. These differences reflect cross‐national variation in long‐term care system design, the accessibility and affordability of institutional care, and cultural expectations regarding family caregiving responsibilities.

While cultural norms and filial piety influence the preference for co‐residence, home‐based care for older adults in China also encounters a series of challenges, including limited policy support, caregiver burden, insufficient recognition of family caregivers, and inadequate community health service capacity [35]. These structural constraints, together with the high prevalence of physical disability, underscore the central role of families in aged care and the need for supportive policies.

### 4.1 Policy Implications

The findings of this study underscore the potential importance of routine, community‐based monitoring of physical function, particularly ADLs, which may help to facilitate early intervention, support independent living, and reduce unnecessary shifts in living arrangements. Functional disability, especially ADL limitations, is a core eligibility criterion in China's Long‐Term Care Insurance (LTCI) system, and timely detection of decline can facilitate access to formal care [49]. With the expansion of LTCI pilot programs, improved availability of formal care may help reduce reliance on co‐residence, complementing family caregiving and supporting ageing in place. Our findings reaffirm the central role of families in aged care, especially in the context of functional decline. Policy priorities should include strengthening community‐based services and expanding LTCI. Key areas for action involve scaling up home‐ and community‐based care, enhancing caregiver support, and implementing programs that preserve or restore functional capacity.

The observed urban–rural disparities in functional transitions and living arrangement changes also call for context‐specific strategies. Long‐Term Care Insurance pilots are concentrated in urban areas, whereas rural regions face more limited coverage and service availability [49], which may reinforce continued reliance on co‐residence following functional loss. Addressing these disparities in LTCI coverage and service provision is critical for ensuring equitable access to long‐term care and reducing unequal caregiving burdens across regions. These strategies should align with China's ‘home‐based, community‐supported, institution‐supplemented’ aged care model to create an integrated, equitable system of ageing support [50].

### 4.2 Limitations

This study has several limitations. First, both ADL and IADL measures are based on self‐reports, which may be subject to recall bias or social desirability bias. Second, due to the relatively small number of institutionalised older adults (*n* = 239), we combined those living alone (*n* = 2205) and those in institutions into a single ‘not with household member (s)’ category. While this approach is justified by the low prevalence of institutional care in China [33], we acknowledge that it may mask meaningful heterogeneity in living arrangements; for example, living with a spouse only differs from living with adult children or in multigenerational households in terms of caregiving dynamics. Future studies with larger samples or more recent waves of the CLHLS could examine transitions among more specific living arrangement states. Third, as an observational study, causal inferences cannot be made, and the relationship between functional decline and living arrangements may be bidirectional. Specifically, while functional decline may trigger living arrangement transitions, the new living environment and its associated social support may also influence subsequent functional trajectories. Fourth, this study is limited by the use of secondary data. Although our models adjusted for a range of important covariates, unmeasured confounding remains possible. Variables such as social support, healthcare accessibility and family structure characteristics, including number of sons versus daughters, children's proximity and caregiving availability, were not consistently available across waves and may have influenced the relationship between functional changes and living arrangements. Fifth, participants included in the analytic sample differed from those excluded in several respects: They were younger, more likely to be male, married, better educated, urban residents and more physically active, while distributions of living arrangements were broadly comparable. These differences may reflect potential selection related to survival and continued participation, which is inherent in longitudinal studies, and should be considered when interpreting the findings.

## 5 Conclusions

The empirical evidence from this study underscores the dynamic interplay between functional health transitions and changes in living arrangements among older adults in China. Functional decline shows a strong association with transitions to co‐residential living, particularly among rural residents. These findings highlight the urgent need to strengthen home‐based and community‐supported care systems, especially in under‐resourced rural areas. As China's ageing population continues to grow and the number of young people declines, thereby challenging the traditional family‐centred care model, policies should prioritise accessible, affordable, and comprehensive care options that support older adults' preferences to age in place. Investing in localised support services will be essential to promoting ageing with dignity across diverse residential settings.

## Funding

The authors have nothing to report.

## Ethics Statement

The Chinese Longitudinal Healthy Longevity Survey had received prior ethical approval from the Institutional Review Boards (IRBs) of Duke University and Peking University (Approval No. IRB00001052‐13074). As this current study involved secondary analysis, it was exempt from IRB approval.

## Consent

The authors have nothing to report.

## Conflicts of Interest

The authors declare no conflicts of interest.

## Data Availability

The data that support the findings of this study are openly available in CLHLS at https://agingcenter.duke.edu/CLHLS.

## Appendix A

**TABLE A1:** Brief introduction of included variables for this current study.

Variable	Classification	Brief description
*Demographic variables*		
Age	Numerical	Interviewee's validated age
Age group	Categorical	Interviewee's validated age
	(0) 65–80 [Reference]	
	(1) 81–95	
	(2) > 95	
Gender	Categorical	Gender as reported by the interviewee
	(0) Female [Reference]	
	(1) Male	
Year of education	Categorical	Formal education received (in years) as reported by the interviewee
	(0) No [Reference]	
	(1) Yes	
Marital status	Categorical	Current marital status as reported by the interviewee
	(0) Others [Reference]	
	(1) Married	
Community of residence	Categorical	Current residential area as reported by the interviewee
	(0) Rural [Reference]	
	(1) Urban	
Number of times suffering from chronic diseases that required inpatient treatment in the past 2 years	Categorical	Number of occurrences of serious illness in the past 2 years, as reported by the interviewee
	(0) None [Reference]	
	(1) 1–2	
	(2) > 2	
Life satisfaction	Numerical	Quality of life as reported by the interviewee
Exercise status	Categorical	Current exercise status as reported by the interviewee
	(0) No [Reference]	
	(1) Yes	
Smoking status	Categorical	Current smoking experience as reported by the interviewee
	(0) No [Reference]	
	(1) Yes	
Alcohol consumption	Categorical	Current alcohol consumption experience as reported by the interviewee
	(0) No [Reference]	
	(1) Yes	
Wave	Categorical	Survey wave in which the interviewees participated
	(0)2011–2012 [Reference]	
	(1) 2014	
	(2) 2017–2018	
*Main predictor*		
Activities of daily living (ADL, six items)	Categorical	Based on self‐reported difficulty with any of the following items:(1) bathing, (2) dressing, (3) eating, (4) indoor transferring, (5) toileting, and (6) continence. Participants with no difficulty in all six are classified as ‘no impairment’; otherwise, as ‘impairment.’
	(0) No impairment [Reference]	
	(1) Impairment	
Instrumental activities of daily living (IADL, eight items)	Categorical	Based on self‐reported ability to perform the following 8 tasks: (1) visiting neighbours, (2) shopping, (3) cooking, (4) washing clothes, (5) walking 1 km, (6) carrying 5 kg, (7) crouching and standing three times, and (8) taking public transportation. Participants reporting no difficulty in all tasks are classified as ‘no difficulty’; otherwise, as ‘difficulty.’
	(0) No difficulty [Reference]	
	(1) Difficulty	
*Outcome variable*		
Living arrangement	Categorical	Living arrangement as reported by the interviewee
	(0) Not with household member (s) [Reference]	
	(1) With household member (s)	

**TABLE A2:** Sociodemographic characteristics of excluded and included Chinese older adults: CLHLS 2008–2018.

	Participants		*p*
	Excluded	Included	
	*n* (%)	*n* (%)	
Living arrangement			0.05
Not with household member (s)	4810 (18)	2444 (19)	
With household member (s)	21,016 (80)	10,116 (81)	
Unknown	317 (1)		
Age (in years)			< 0.001
Less than 65	474 (2)		
65–80	6918 (26)	4970 (40)	
81–95	10,271 (39)	5641 (45)	
> 95	8472 (32)	1949 (16)	
Unknown	8 (0)		
Gender			< 0.001
Female	15,346 (59)	6584 (52)	
Male	10,797 (41)	5976 (48)	
Unknown			
Year of education (in years)			< 0.001
No	14,803 (57)	6739 (54)	
Yes	10,248 (39)	5821 (46)	
Unknown	1092 (4)		
Marital status			< 0.001
Others	17,440 (67)	7304 (58)	
Married	8378 (32)	5256 (42)	
Unknown	325 (1)		
Community of residence			< 0.001
Rural	13,804 (53)	5934 (47)	
Urban	12,339 (47)	6626 (53)	
Unknown			
Smoking status			< 0.001
No	22,139 (85)	10,221 (81)	
Yes	3711 (14)	2339 (19)	
Unknown	293 (1)		
Alcohol consumption status			< 0.001
No	22,053 (84)	10,411 (83)	
Yes	3686 (14)	2149 (17)	
Unknown	404 (2)		
Exercise status			< 0.001
No	19,263 (74)	8111 (65)	
Yes	6439 (25)	4449 (35)	
Unknown	441 (2)		
Number of times suffering from chronic diseases that required inpatient treatment in the past 2 years			< 0.001
None	19,604 (75)	9498 (76)	
1–2	4362 (17)	2618 (21)	
> 2	1224 (5)	444 (4)	
Unknown	953 (4)		
Life satisfaction (mean, SD)	3.77 (0.81)	3.79 (0.82)	0.10
Unknown	3838 (15)		
Wave			< 0.001
2008–2009	9331 (36)		
2011–2012	1954 (7)	6965 (55)	
2014	1702 (7)	4613 (37)	
2017–2018	13,156 (50)	982 (8)	

*Note:* The excluded sample included 26,143 observations from 23,657 participants, and the analytic sample included 12,560 observations from 7873 participants. Participants were required to have data from at least two adjacent waves to construct changes in ADL and IADL physical disability. *P* values were obtained using *t*‐test for continuous variables and Pearson's χ^2^ tests for categorical variables.

**TABLE A3:** Interaction between changes in functional disability and community of residence on living arrangements among Chinese older adults: Evidence from the CLHLS, 2008–2018.

	Change of living arrangement	
	From not with household member (s) to with household member (s)	From with household member (s) to with household member (s)
	HR (95% CI)	HR (95% CI)
ADL transition		
No change		
Impairment to no impairment (Improved)	0.85 (0.70, 1.04)	1.08 (0.89, 1.30)
No impairment to impairment (Declined)	1.15 (1.06, 1.25)***	1.25 (1.14, 1.38)***
IADL transition		
No change		
Difficulty to no difficulty (Improved)	0.97 (0.88, 1.08)	1.01 (0.87, 1.17)
No difficulty to difficulty (Declined)	0.98 (0.91, 1.05)	1.03 (0.94, 1.13)
Age (in years)		
65–80		
81–95	1.12 (1.07, 1.18)***	1.08 (1.02, 1.15)**
> 95	1.56 (1.45, 1.69)***	1.41 (1.3, 1.54)***
Gender		
Female		
Male	0.89 (0.84, 0.93)***	0.93 (0.87, 0.99)*
Education (in years)		
No		
Yes	1.05 (1.00, 1.10)	1.03 (0.97, 1.09)
Marital status		
Others		
Married	2.36 (2.24, 2.48)***	1.68 (1.58, 1.80)***
Community of residence		
Rural		
Urban	0.96 (0.91, 1.01)	1.1 (1.03, 1.17)**
Smoking status		
No		
Yes	1.02 (0.97, 1.08)	0.99 (0.92, 1.06)
Alcohol consumption status		
No		
Yes	1.01 (0.95, 1.06)	0.95 (0.88, 1.03)
Exercise status		
No		
Yes	0.96 (0.92, 1.00)	0.99 (0.94, 1.05)
Number of times suffering from chronic diseases that required inpatient treatment in the past 2 years		
None		
1–2	1.02 (0.97, 1.07)	1.08 (1.02, 1.14)**
> 2	1.03 (0.93, 1.15)	1.04 (0.93, 1.16)
Life satisfaction	1.08 (1.06, 1.11)***	1.07 (1.04, 1.11)***
ADL transition*residence		
Impairment to no impairment: residence urban	1.13 (0.86, 1.48)	0.76 (0.58, 0.99)
No impairment to impairment: residence urban	1.02 (0.91, 1.13)	0.79 (0.69, 0.90)***
IADL transition*residence		
Residence urban: IADL difficulty to no difficulty	1.05 (0.91, 1.21)	0.93 (0.77, 1.13)
Residence urban: IADL no difficulty to difficulty	1.03 (0.93, 1.13)	0.96 (0.85, 1.08)

*Note:* **p* < 0.05; ***p* < 0.01; ****p* < 0.001.

Abbreviations: CI, confidence interval; HR, hazard ratio.

**TABLE A4:** (1) Sensitivity analysis of functional disability classification (two or more impairments/difficulties) and its association with living arrangements among Chinese older adults: CLHLS, 2008–2018. (2) Sensitivity analysis of the interaction between changes in functional disability (two or more impairments/difficulties) and community residence on living arrangements among Chinese older adults: Evidence from the CLHLS, 2008–2018. (3) Sensitivity analysis of associations between changes in functional disability (two or more impairments/difficulties) and living arrangements among Chinese older adults, stratified by community of residence: Evidence from the CLHLS, 2008–2018.

1	Change in living arrangement	
	From not with household member (s) to with household member (s)	From with household member (s) to with household member (s)
	HR (95% CI)	HR (95% CI)
ADL transition		
No change		
Impairment to no impairment (Improved)	1.02 (0.93,1.13)	1.01 (0.94,1.1)
No impairment to impairment (Declined)	1.12 (1.09,1.16)***	1.05 (1.01,1.08)**
IADL transition		
No change		
Difficulty to no difficulty (Improved)	0.95 (0.91,0.99)*	0.95 (0.9,1)*
No difficulty to difficulty (Declined)	1 (0.97,1.02)	1.01 (0.98,1.04)
Age (in years)		
65–80		
81–95	1.08 (1.05,1.12)***	1.04 (1.01,1.07)**
> 95	1.29 (1.24,1.35)***	1.15 (1.11,1.2)***
Gender		
Female		
Male	0.93 (0.9,0.96)***	0.97 (0.94,1)*
Education (in years)		
No		
Yes	1.02 (1,1.05)	1.01 (0.98,1.03)
Marital status		
Others		
Married	1.57 (1.52,1.62)***	1.24 (1.2,1.28)***
Community of residence		
Rural		
Urban	0.98 (0.96,1.01)	1.01 (0.99,1.04)
Smoking status		
No		
Yes	1.01 (0.98,1.04)	1 (0.97,1.03)
Alcohol consumption status		
No		
Yes	1.01 (0.98,1.04)	0.98 (0.95,1.01)
Exercise status		
No		
Yes	0.98 (0.96,1.01)	1 (0.97,1.02)
Number of times suffering from chronic diseases that required inpatient treatment in the past 2 years		
None		
1–2	1.01 (0.98,1.04)	1.03 (1,1.05)*
> 2	1 (0.95,1.06)	1.01 (0.96,1.06)
Life satisfaction	1.05 (1.04,1.06)***	1.03 (1.02,1.04)***

2	Change in living arrangement	
	From not with household member (s) to with household member (s)	From with household member (s) to with household member (s)
	HR (95% CI)	HR (95% CI)
ADL transition		
No change		
Impairment to no impairment (Improved)	1.05 (0.92,1.21)	1.08 (0.99,1.19)
No impairment to impairment (Declined)	1.14 (1.08,1.2)***	1.08 (1.03,1.14)**
IADL transition		
No change		
Difficulty to no difficulty (Improved)	0.94 (0.88,1)*	0.94 (0.87,1.02)
No difficulty to difficulty (Declined)	1 (0.96,1.04)	0.99 (0.95,1.04)
Age (in years)		
65–80		
81–95	1.08 (1.05,1.12)***	1.04 (1.01,1.07)**
> 95	1.29 (1.24,1.35)***	1.15 (1.11,1.2)***
Gender		
Female		
Male	0.93 (0.9,0.96)***	0.97 (0.94,1)*
Education (in years)		
No		
Yes	1.02 (1,1.05)	1.01 (0.98,1.03)
Marital status		
Others		
Married	1.57 (1.52,1.62)***	1.24 (1.2,1.28)***
Community of residence		
Rural		
Urban	0.98 (0.96,1.01)	1.01 (0.99,1.04)
Smoking status		
No		
Yes	1.01 (0.98,1.04)	1 (0.97,1.03)
Alcohol consumption status		
No		
Yes	1.01 (0.98,1.04)	0.98 (0.95,1.01)
Exercise status		
No		
Yes	0.98 (0.96,1.01)	1 (0.97,1.02)
Number of times suffering from chronic diseases that required inpatient treatment in the past 2 years		
None		
1–2	1.01 (0.98,1.04)	1.03 (1,1.05)*
> 2	1 (0.95,1.06)	1.01 (0.96,1.06)
Life satisfaction	1.05 (1.04,1.06)***	1.03 (1.02,1.04)***
ADL transition*Residence		
Impairment no impairment: Residence urban	0.95 (0.79,1.15)	0.91 (0.79,1.04)
No impairment to impairment: Residence urban	0.98 (0.91,1.04)	0.94 (0.89,1.01)
IADL transition*Residence		
Residence Urban: IADL difficulty to no difficulty	1.03 (0.94,1.12)	1.01 (0.92,1.12)
Residence Urban: IADL no difficulty to difficulty	1 (0.95,1.06)	1.03 (0.98,1.09)

3	Change in living arrangement among urban residents		Change in living arrangement among rural residents	
	From not with household member (s) to with household member (s)	From with household member (s) to with household member (s)	From not with household member (s) to with household member (s)	From with household member (s) to with household member (s)
	HR (95% CI)	HR (95% CI)	HR (95% CI)	HR (95% CI)
ADL transition				
No change				
Impairment to no impairment (Improved)	0.97 (0.86,1.1)	1.04 (0.96,1.13)	1.07 (0.95,1.21)	1.05 (0.9,1.23)
No impairment to impairment (Declined)	1.1 (1.06,1.15)***	1.01 (0.96,1.07)	1.14 (1.08,1.19)***	1.11 (1.05,1.17)***
IADL transition				
No change				
Difficulty to no difficulty (Improved)	0.95 (0.9,1.01)	0.97 (0.91,1.04)	0.92 (0.87,0.98)**	0.97 (0.88,1.06)
No difficulty to difficulty (Declined)	0.99 (0.96,1.03)	1.02 (0.98,1.06)	0.99 (0.96,1.03)	0.99 (0.94,1.04)
Age (in years)				
65–80				
81–95	1.08 (1.05,1.12)***	1.03 (1,1.07)	1.08 (1.04,1.12)***	1.04 (0.99,1.09)
> 95	1.27 (1.21,1.34)***	1.16 (1.1,1.22)***	1.3 (1.23,1.37)***	1.12 (1.04,1.2)**
Gender				
Female				
Male	0.93 (0.9,0.97)***	0.96 (0.92,1)*	0.93 (0.9,0.97)***	0.97 (0.92,1.03)
Education (in years)				
No				
Yes	1.03 (1,1.07)	1 (0.96,1.03)	1.02 (0.99,1.06)	1 (0.95,1.04)
Marital status				
Others				
Married	1.51 (1.45,1.57)***	1.23 (1.18,1.29)***	1.63 (1.56,1.69)***	1.23 (1.17,1.3)***
Smoking status				
No				
Yes	1.02 (0.98,1.06)	1 (0.96,1.04)	1.01 (0.97,1.05)	1 (0.95,1.05)
Alcohol consumption status				
No				
Yes	1 (0.96,1.04)	0.98 (0.94,1.03)	1.02 (0.98,1.06)	0.98 (0.93,1.04)
Exercise status				
No				
Yes	0.98 (0.95,1.01)	1.01 (0.98,1.04)	0.98 (0.95,1.01)	0.99 (0.94,1.03)
Number of times suffering from chronic diseases that required inpatient treatment in the past 2 years				
None				
1–2	1.02 (0.99,1.06)	1.01 (0.98,1.05)	1.03 (0.99,1.06)	1.01 (0.97,1.06)
> 2	1.05 (0.98,1.12)	1.02 (0.96,1.09)	1 (0.91,1.09)	0.99 (0.88,1.1)
Life satisfaction	1.04 (1.03,1.06)***	1.02 (1.01,1.04)**	1.06 (1.04,1.08)***	1.03 (1.01,1.06)*

*Note:* **p* < 0.05; ***p* < 0.01; ****p* < 0.001.

Abbreviations: CI, confidence interval; HR, hazard ratio.
